# Association between serum lipoprotein(a) levels and aortic vulnerable plaques assessed by non-obstructive general angioscopy

**DOI:** 10.1016/j.athplu.2026.100603

**Published:** 2026-09-12

**Authors:** Keisuke Kojima, Yudai Tanaka, Katsunori Fukumoto, Yasunari Ebuchi, Tokio Nishiwaki, Naoki Kurito, Satoaki Tomimatsu, Yuta Hotsubo, Tetsuro Nagao, Ran Sumida, Saki Mizobuchi, Shohei Migita, Masatsugu Miyagawa, Koichiro Hori, Yutaka Koyama, Riku Arai, Suguru Migita, Mitsumasa Sudo, Daisuke Fukamachi, Yasuo Okumura

**Affiliations:** Division of Cardiology, Department of Medicine, Nihon University School of Medicine, Tokyo, Japan

**Keywords:** Lipoprotein(a), Plaque rupture, Aortic atherosclerosis, Vulnerable plaque, Non-obstructive general angioscopy

## Abstract

**Background and aims:**

Elevated lipoprotein(a) [Lp(a)] levels are recognized as a genetic risk factor for atherosclerotic cardiovascular disease. Previous coronary imaging studies have linked elevated Lp(a) levels to vulnerable coronary plaque characteristics. However, the relationship between elevated Lp(a) levels and vulnerable aortic plaque characteristics remains unclear. We investigated the association between serum Lp(a) levels and vulnerable aortic plaque characteristics assessed by non-obstructive general angioscopy (NOGA).

**Methods:**

We analyzed 276 consecutive patients with coronary artery disease who underwent NOGA between December 2014 and March 2023. Patients were divided according to serum Lp(a) levels using a 30 mg/dL cutoff. Vulnerable aortic plaque characteristics, including plaque rupture, thrombus, yellow plaque, ulceration, and fissure, were assessed by NOGA.

**Results:**

Elevated Lp(a) levels (>30 mg/dL) were observed in 27% of patients, whereas markedly elevated Lp(a) levels (>50 mg/dL) were observed in 14%. Patients with elevated Lp(a) levels showed a significantly higher prevalence of plaque rupture (84% vs. 62%, p < 0.001), thrombus, and fissure than those with lower Lp(a) levels. In contrast, the prevalence of yellow plaque did not differ significantly between groups. The prevalence of plaque rupture progressively increased according to Lp(a) strata. In multivariate logistic regression analysis, elevated Lp(a) levels remained independently associated with plaque rupture after adjustment for traditional cardiovascular risk factors.

**Conclusions:**

Elevated serum Lp(a) levels were independently associated with aortic plaque rupture assessed by NOGA. These findings suggest that elevated Lp(a) levels may be associated with persistent aortic plaque vulnerability despite intensive LDL-C lowering.

## Highlights

• Elevated Lp(a) was associated with aortic plaque rupture and thrombus.
• Aortic plaque rupture frequency increased across Lp(a) categories.
• Lp(a) was independently associated with aortic plaque rupture.
• Associations persisted despite LDL-C levels <55 mg/dL.
• Elevated Lp(a) may contribute to plaque destabilization rather than plaque burden.

## Graphical abstract

**Figure:**
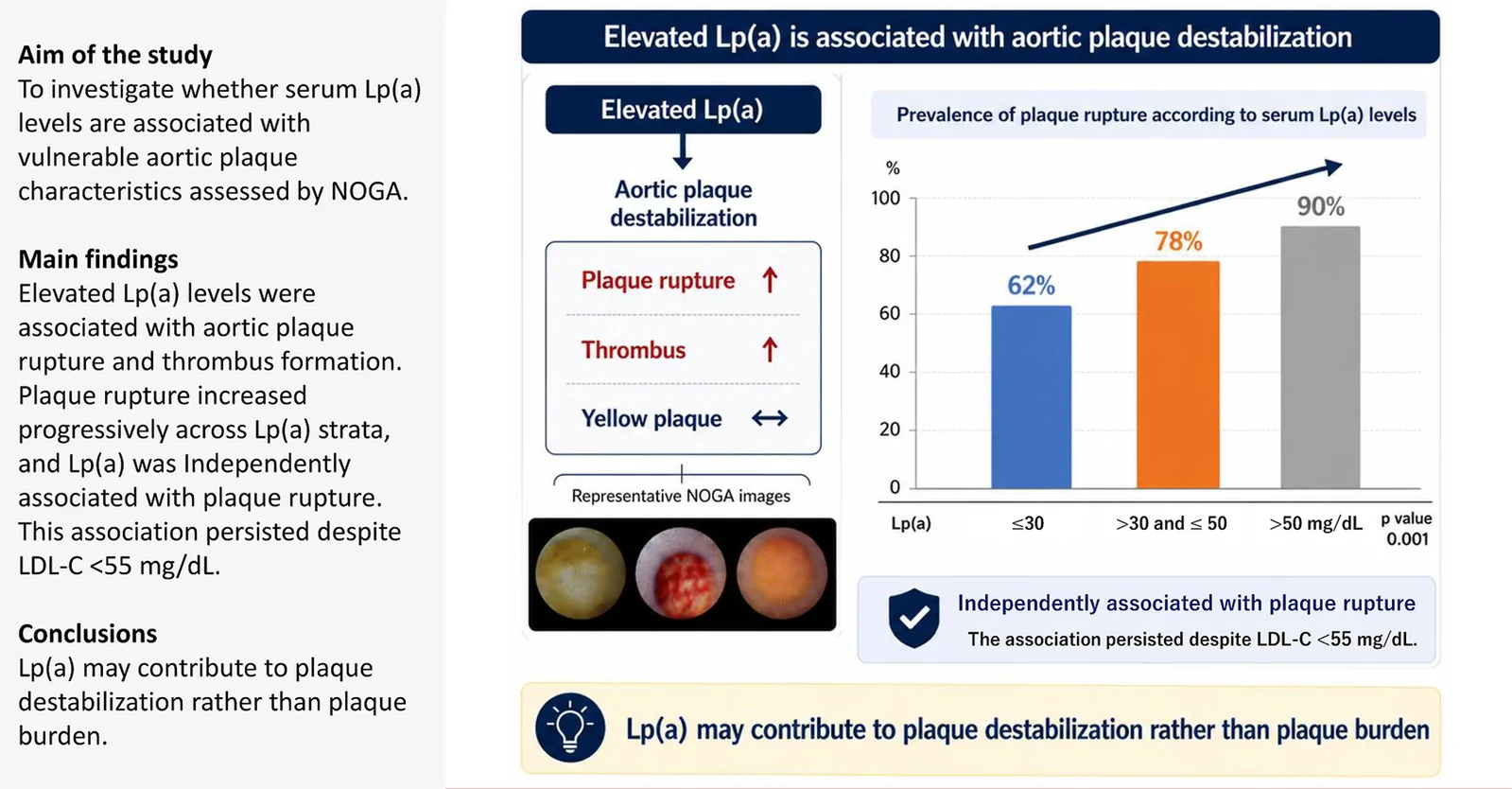

## 1 Introduction

Elevated lipoprotein(a) [Lp(a)] levels are recognized as a genetically determined risk factor for atherosclerotic cardiovascular disease (ASCVD) [1,2]. Current American and European guidelines recommend measurement of Lp(a) levels for cardiovascular risk stratification [3,4]. Elevated Lp(a) levels have been associated with myocardial infarction, ischemic stroke, peripheral artery disease, and cardiovascular death [[5], [6], [7], [8]].

Beyond its established association with cardiovascular events, previous coronary imaging studies have suggested relationships between elevated Lp(a) levels and vulnerable coronary plaque characteristics [9].

In contrast, previous studies evaluating the relationship between Lp(a) and aortic atherosclerosis have primarily focused on plaque burden, aortic atheroma severity, or vascular calcification. Whether elevated Lp(a) levels are associated with vulnerable aortic plaque characteristics and plaque rupture remains unclear. Non-obstructive general angioscopy (NOGA) enables direct visualization of the aortic luminal surface and allows assessment of vulnerable plaque morphologies in vivo [10,11]. Previous studies have also demonstrated associations between vulnerable aortic plaques assessed by NOGA and future cardiovascular events [12].

Because Lp(a) has been implicated in plaque vulnerability in coronary imaging studies, we hypothesized that elevated Lp(a) levels would be associated with vulnerable aortic plaque characteristics, particularly plaque rupture. Therefore, we investigated the association between serum Lp(a) levels and angioscopically defined aortic plaque characteristics in patients with coronary artery disease.

## 2 Materials and methods

### 2.1 Study design and subjects

This was a single-center, observational imaging study of 430 consecutive patients with CAD who underwent NOGA for the aorta at Nihon University Itabashi Hospital between December 2014 and March 2023. Patients were eligible for inclusion if they were aged over 20 years and underwent coronary angiography or percutaneous coronary intervention (PCI). Patients were referred for NOGA evaluation at the discretion of the attending physicians during clinically indicated cardiac catheterization procedures.

Exclusion criteria were identical to those of our previous study, including the use of mechanical circulatory support, hemodynamic instability (ie, Killip class >2 or cardiogenic shock), acute aortic syndrome, acute-phase cerebral infarction, allergy to contrast media, and pregnancy [13]. Among the 430 patients who underwent NOGA, 154 patients without available serum Lp(a) measurements were excluded. Consequently, 276 patients were included in the present analysis (Supplementary Fig. 1). The study received approval from the institutional review board at Nihon University Itabashi Hospital (RK-210713-7). Written informed consent was obtained from all study participants.

Fasting blood samples were obtained before catheterization. Serum Lp(a) concentrations were measured in mg/dL using a turbidimetric immunoassay (N-Assay TIA Lp(a) Nittobo; Nittobo Medical Co., Tokyo, Japan). The analytical performance and harmonization of this assay against the IFCC-endorsed reference measurement procedure have been previously reported [14].

### 2.2 Cardiac catheterization and angioscopic system

Cardiac catheterization was performed via either the radial or femoral artery according to standard institutional practice, as previously described [12]. Following coronary angiography and, when indicated, PCI, NOGA was performed using a VISIBLE Fiber (FT-203F, Fiber Tech Co. Ltd., Tokyo, Japan) or Smart-I Fiber (S8-6k, Amphi Co. Ltd., Osaka, Japan) connected to a dedicated console (InterTec Medicals Co. Ltd., Osaka, Japan).

To obtain a clear visual field, low-molecular-weight dextran and lactated Ringer's solution (Low Molecular Dextran L Injection; Otsuka Pharmaceutical Factory, Tokushima, Japan) were continuously infused through the catheter system, thereby displacing blood from the observation site. Angioscopic images were digitally recorded and stored for subsequent offline analysis. The total volume of infused solution during each examination was approximately 50–80 mL.

The entire aorta, extending from the ascending aorta to the terminal aorta, was systematically examined. A 4-Fr probing catheter and the angioscopic fiber were advanced through a 6-Fr Ikari Left 3.5 guiding catheter (Goodman, Nagoya, Japan). During image acquisition, the catheter system was gradually withdrawn while being rotated, allowing circumferential visualization of the aortic luminal surface throughout the thoracic and abdominal aorta.

### 2.3 Assessment of non-obstructive general angioscopy

**Fig. 1:**
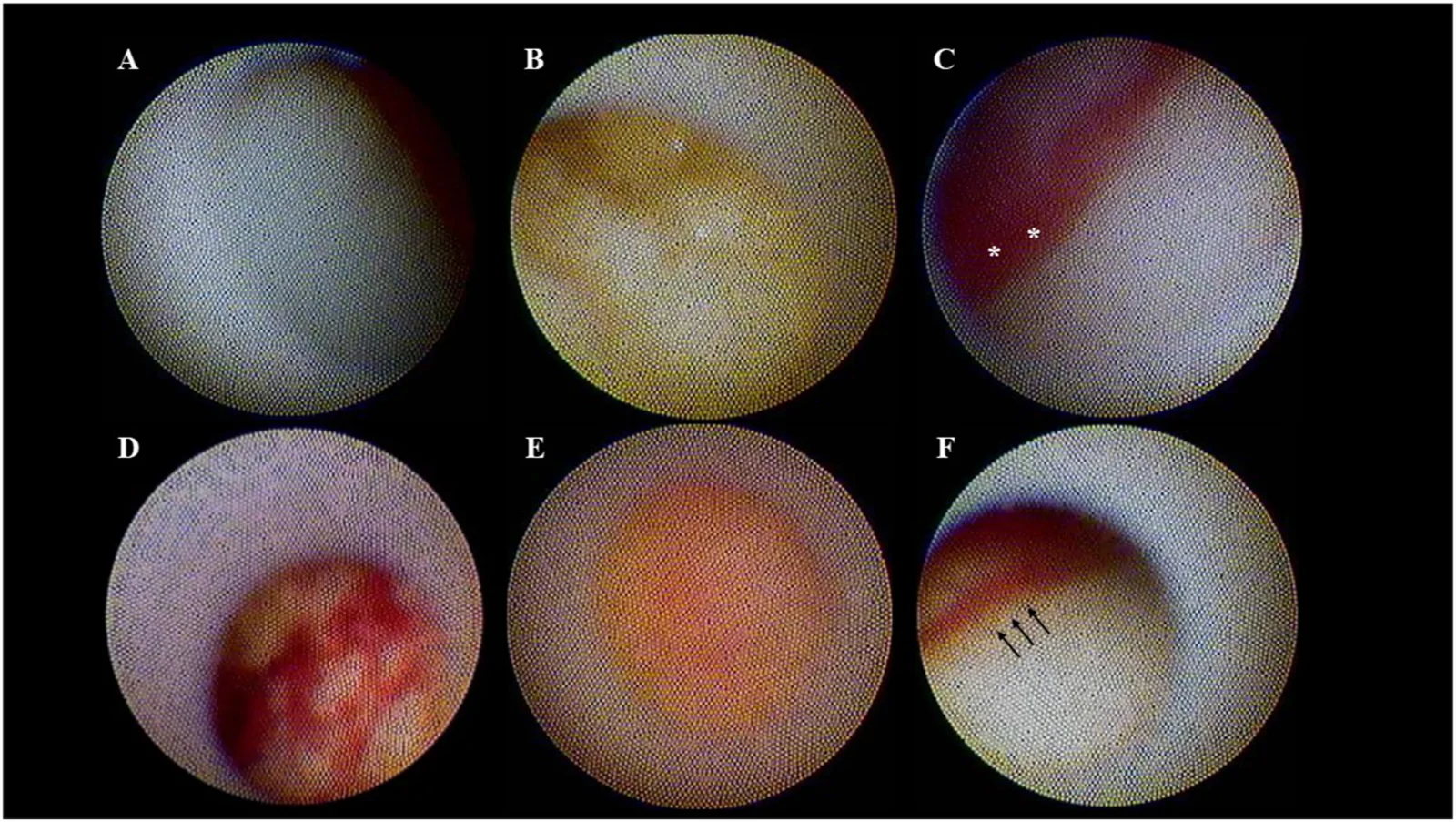
Representative NOGA findings Representative angioscopic findings observed by NOGA. (A) Normal intima. (B) Plaque rupture. (C) Thrombus (*). (D) Ulceration. (E) Intense yellow plaque (yellow grade 3). (F) Fissure (arrows). NOGA, non-obstructive general angioscopy.

Aortic plaque refers to observable atherosclerosis affecting the inner surface of the aorta [[10], [11], [12], [13]]. NOGA was performed to assess the entire aorta, including the ascending, thoracic, abdominal, and terminal aorta. Characteristics of the plaque were documented, including plaque rupture, thrombi, yellow plaque, ulcer, and fissure. Representative angioscopic findings observed by NOGA are shown in Fig. 1. Yellow color grade (yellow grade) was defined as 0 (white), 1 (light yellow), 2 (yellow), or 3 (intense yellow) [15]. The presence of yellow plaques was defined as a maximum yellow color grade of 2 or 3 [11,13]. High levels of consistency among proficient observers have been confirmed in the analysis of NOGA [11,12,16,17].

### 2.4 Primary outcome

The primary outcome of interest was the presence of aortic plaque rupture assessed by NOGA. Secondary outcomes included the presence of thrombus, fissure, ulceration, and yellow plaque, as well as the number of each plaque morphology throughout the aorta.

### 2.5 Statistical analysis

Continuous variables were reported as mean ± standard deviation for normally distributed data or median with interquartile range (IQR) for non-normally distributed data. Categorical variables were presented as counts and percentages. Comparisons of continuous variables were performed using Student's t-test for normally distributed data or Mann–Whitney *U* test for non-normally distributed data. Categorical variables were presented as counts and percentages and analyzed using the chi-squared test or Fisher's exact test, as appropriate. Patients were primarily categorized according to a serum Lp(a) cutoff value of 30 mg/dL (≤30 vs > 30 mg/dL), based on previous epidemiological studies and clinical practice in Japan. In addition, sensitivity analyses were performed using a cutoff value of 50 mg/dL (≤50 vs > 50 mg/dL). As an exploratory analysis, we also examined whether the association between elevated Lp(a) levels and vulnerable plaque characteristics persisted among patients who achieved LDL-C levels <70 mg/dL. An additional exploratory analysis was performed among patients who achieved LDL-C levels <55 mg/dL. Univariate logistic regression analyses were performed to examine factors associated with plaque rupture. Multivariable logistic regression analysis was subsequently performed to evaluate the independent association between elevated Lp(a) levels and plaque rupture. The multivariable model included age, sex, diabetes mellitus, hypertension, smoking history, low-density lipoprotein cholesterol (LDL-C), chronic kidney disease, high-sensitivity C-reactive protein (hs-CRP), and elevated Lp(a) levels (>30 mg/dL), selected based on established cardiovascular risk factors and biological plausibility. Additional sensitivity analyses were performed by replacing the >30 mg/dL cutoff with >50 mg/dL and by entering Lp(a) as a continuous variable. To evaluate whether the association between elevated Lp(a) levels and plaque rupture differed according to sex, an interaction analysis between sex and elevated Lp(a) was additionally performed. Odds ratios (ORs) and 95% confidence intervals (CIs) were calculated. All statistical analyses, including the multivariate logistic regression analysis, were performed using JMP Pro software version 16.1.0 (SAS Institute, Cary, USA). A p-value < 0.05 was considered statistically significant.

## 3 Results

### 3.1 Patient characteristics

A total of 430 consecutive patients underwent NOGA during the study period. After exclusion of 154 patients without available serum Lp(a) measurements, 276 patients were included in the final analysis (Supplementary Fig. 1). Among them, 75 patients (27%) had elevated Lp(a) levels >30 mg/dL, whereas 201 patients had Lp(a) levels ≤30 mg/dL (Supplementary Fig. 2). In addition, 14% of patients showed markedly elevated Lp(a) levels >50 mg/dL. Patients with elevated Lp(a) levels (>30 mg/dL) were more likely to be male and had a higher prevalence of old myocardial infarction, peripheral artery disease, and polyvascular disease compared with patients with lower Lp(a) levels (≤30 mg/dL). Use of ezetimibe and renin–angiotensin system inhibitors was also more frequent in the high Lp(a) group. No significant differences were observed in LDL-cholesterol, HDL-cholesterol, triglyceride levels, or HbA1c between the two groups.

### 3.2 Association between Lp(a) levels and aortic plaque characteristics

**Fig. 2:**
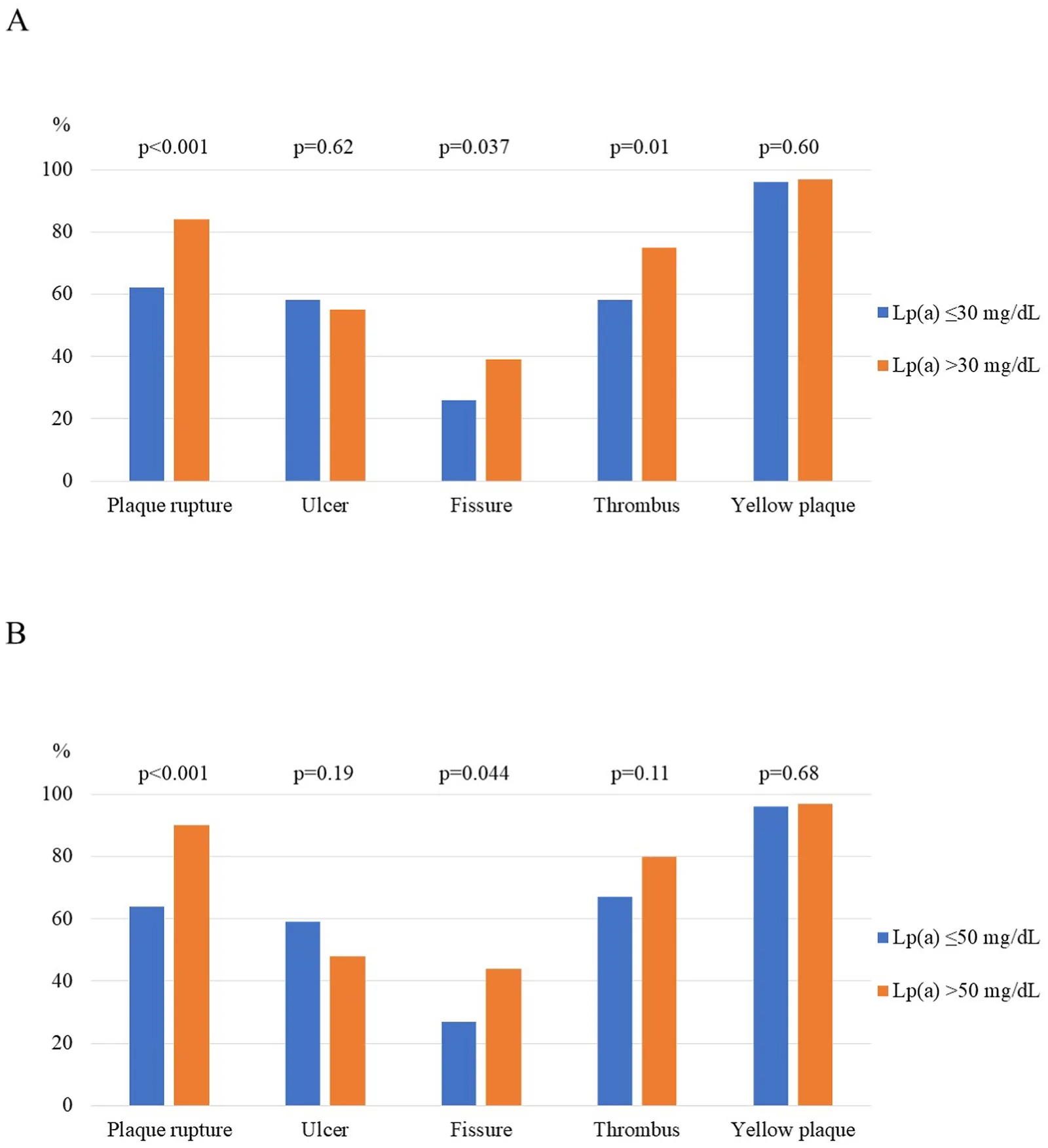
A: Association between plaque characteristics according to serum Lp(a) levels (≤30 vs > 30 mg/dL) Elevated serum Lp(a) levels (>30 mg/dL) are associated with a higher prevalence of plaque rupture, thrombus, and fissure compared with lower Lp(a) levels (≤30 mg/dL). In contrast, the prevalence of yellow plaque does not differ significantly between the two groups. B: Association between plaque characteristics according to serum Lp(a) levels (≤50 vs > 50 mg/dL) Elevated serum Lp(a) levels (>50 mg/dL) are associated with a higher prevalence of plaque rupture and fissure compared with lower Lp(a) levels (≤50 mg/dL). In contrast, the prevalence of yellow plaque does not differ significantly between the two groups. Lp(a), lipoprotein(a); NOGA, non-obstructive general angioscopy.

Patients with elevated Lp(a) levels showed a significantly higher frequency of plaque rupture compared with those with lower Lp(a) levels (84% vs 62%, p < 0.001). Similarly, fissure and thrombus were more frequently observed in patients with elevated Lp(a) levels. In contrast, the prevalence of yellow plaque did not differ significantly between the two groups (Table 1, Fig. 2A). Regarding the number of plaques, patients with elevated Lp(a) levels had a significantly greater number of plaque ruptures and thrombi. However, the number of yellow plaques was comparable between groups. When a cutoff value of 50 mg/dL was applied, patients with elevated Lp(a) levels also demonstrated a significantly higher prevalence of plaque rupture and fissure than those with lower Lp(a) levels (Fig. 2B).

**Table 1:** Patient characteristics according to Lp(a) levels.

	Lp(a) ≤30 mg/dL	Lp(a) > 30 mg/dL	P value
	n = 201	n = 75	
Age (yrs)	67 ± 12	67 ± 16	0.83
Gender, male	154 (78)	69 (93)	0.003
BMI	24.6 ± 4.0	23.8 ± 3.1	0.12
**Atherosclerotic risk factors**			
Diabetes Mellitus	85 (43)	27 (36)	0.33
Dyslipidemia	150 (75)	62 (83)	0.18
Hypertension	154 (77)	52 (69)	0.19
Smoking history	117 (59)	54 (72)	0.040
Chronic kidney disease	58 (29)	30 (40)	0.078
**Comorbidities**			
Old myocardial infarction	57 (28)	33 (44)	0.015
Ischemic stroke	23 (11)	7 (9)	0.62
Peripheral artery disease	10 (5)	11 (15)	0.011
Multivessel disease	49 (25)	25 (34)	0.13
Polyvascular disease	29 (14)	20 (27)	0.019
**Laboratory data**			
Creatinine (mg/dl)	0.83 [0.72-0.95]	0.87 [0.75-1.12]	0.64
eGFR (ml/min/1.73m^2^)	68.7 ± 20.5	67.5 ± 20.9	0.67
HbA1c (%)	6.3 ± 0.9	6.4 ± 1.3	0.33
HDL-C (mg/dl)	45.6 ± 13.5	46.1 ± 11.2	0.81
LDL-C (mg/dl)	91.6 ± 35.6	92.4 ± 37.2	0.86
Triglyceride (mg/dl)	125 [83-191]	122 [85-156]	0.19
Uric acid (mg/dl)	5.6 ± 1.3	5.8 ± 1.5	0.29
hs-CRP (mg/dl)	0.10 [0.04-0.28]	0.10 [0.05-0.28]	0.50
Lp(a) (mg/dL)	14 [8-21]	52 [37-73]	<0.001
**Medications**			
Aspirin	116 (58)	40 (53)	0.51
Statin	143 (71)	60 (80)	0.13
Ezetimib	33 (16)	23 (31)	0.009
PCSK9-i	2 (1)	2 (3)	0.33
RAS-i	124 (62)	56 (75)	0.041
Beta-blocker	86 (43)	41 (55)	0.063
Oral Hypoglycemic Agents	116 (58)	40 (53)	0.51

**NOGA findings**			
**Frequency of plaque**			
Plaque rupture	125 (62)	63 (84)	<0.001
Ulcer	116 (58)	41 (55)	0.62
Fissure	52 (26)	29 (39)	0.037
Thrombus	117 (58)	54 (75)	0.010
Yellow plaque	192 (96)	72 (97)	0.60
**Number of plaque**			
Plaque rupture	1.9 ± 2.3	3.0 ± 2.8	<0.001
Ulcer	1.0 ± 1.2	1.0 ± 1.2	0.66
Fissure	0.4 ± 0.8	0.6 ± 1.0	0.061
Thrombus	2.2 ± 2.6	3.0 ± 2.8	0.043
Yellow plaque	9.7 ± 4.8	9.8 ± 4.8	0.89

Data are presented as mean ± standard deviation, median [interquartile range], or n (%). BMI, body mass index; eGFR, estimated glomerular filtration rate; HbA1c, hemoglobin A1c; HDL-C, high-density lipoprotein cholesterol; hs-CRP, high-sensitivity C-reactive protein; LDL-C, low-density lipoprotein cholesterol; Lp(a), lipoprotein(a); NOGA, non-obstructive general angioscopy; PCSK9-i, proprotein convertase subtilisin/kexin type 9 inhibitor; RAS-i, renin–angiotensin system inhibitor.

The association between elevated Lp(a) levels and plaque rupture remained evident even among patients with LDL-C levels <70 mg/dL, a recommended target for secondary prevention. Similarly, thrombus formation was more frequently observed in patients with elevated Lp(a) levels despite controlled LDL-C levels (Supplementary Fig. 3 and Supplementary Table 1). Furthermore, the association between elevated Lp(a) levels and plaque rupture persisted among patients achieving LDL-C levels <55 mg/dL (Supplementary Fig. 4).

### 3.3 Incremental association between Lp(a) levels and plaque rupture

**Fig. 3:**
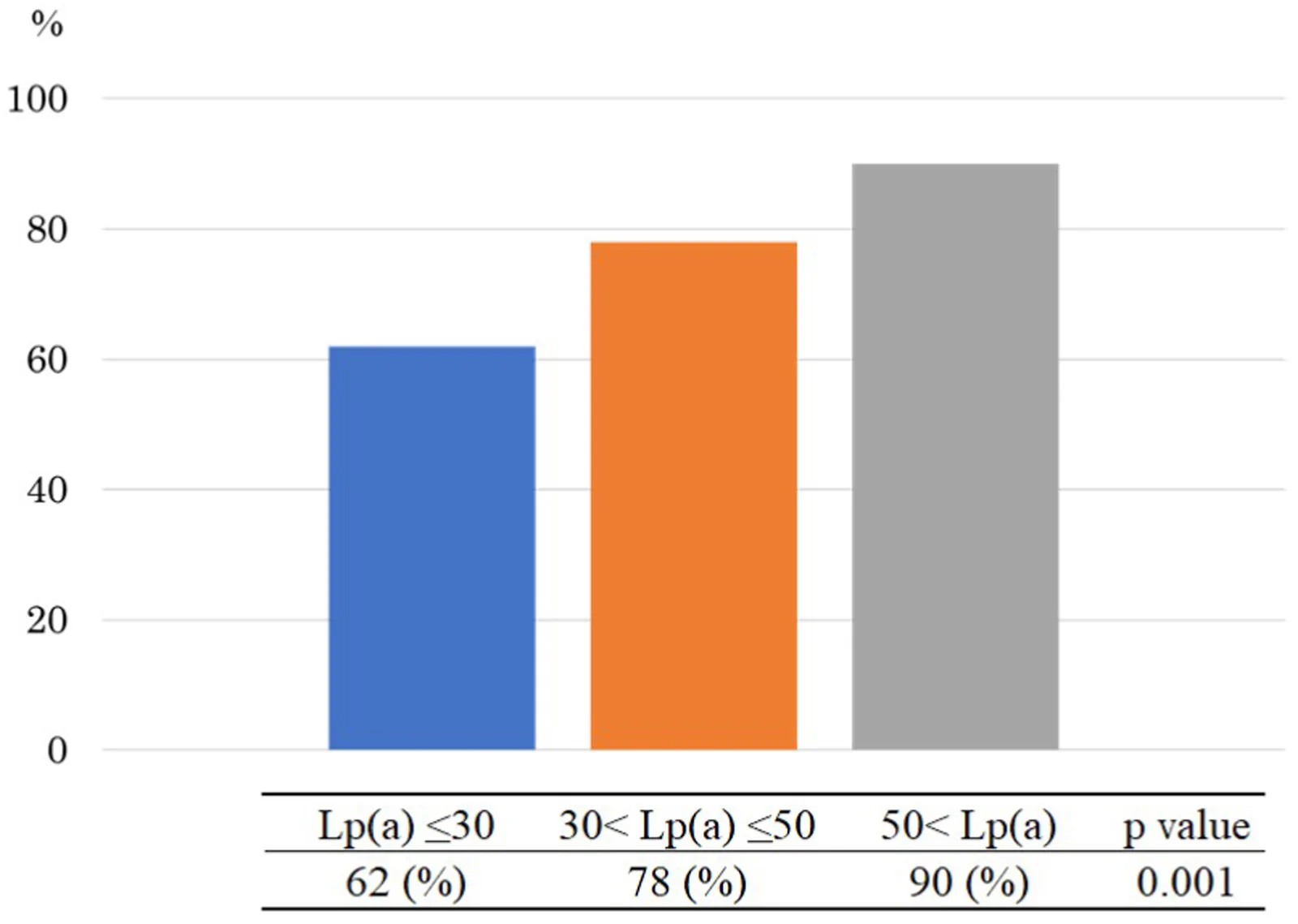
Prevalence of plaque rupture according to serum Lp(a) levels The prevalence of plaque rupture progressively increases with increasing serum Lp(a) levels. Plaque rupture is observed in 62% of patients with Lp(a) levels ≤30 mg/dL, 78% of patients with Lp(a) levels >30 and ≤ 50 mg/dL, and 90% of patients with Lp(a) levels >50 mg/dL. Lp(a), lipoprotein(a).

The frequency of plaque rupture progressively increased according to Lp(a) level strata. Plaque rupture was observed in 62% of patients with Lp(a) ≤30 mg/dL, 78% of patients with Lp(a) levels between >30 and ≤ 50 mg/dL, and 90% of patients with Lp(a) levels >50 mg/dL (p = 0.001) (Fig. 3).

### 3.4 Univariate and multivariate analyses to identify factors associated with plaque rupture

In univariate logistic regression analysis, elevated Lp(a) levels >30 mg/dL were significantly associated with plaque rupture (OR 3.19, 95% CI 1.67–6.56, p < 0.001). After adjustment for traditional cardiovascular risk factors, elevated Lp(a) levels remained independently associated with plaque rupture (OR 3.56, 95% CI 1.63–7.80, p = 0.002) (Table 2). (Table 2). Similar findings were obtained using a cutoff value of >50 mg/dL (Supplementary Table 2) and when serum Lp(a) was analyzed as a continuous variable (Supplementary Table 3). No significant interaction between sex and elevated Lp(a) was observed (P for interaction = 0.69).

**Table 2:** Univariate and multivariate analyses of factors associated with plaque rupture.

Variables	univariate			multivariate		
	OR	95% CI	P value	OR	95% CI	P value
Age (yrs)	1.03	1.01-1.05	0.002	1.07	1.03-1.10	<0.001
Gender, male	1.01	0.51-1.93	0.97	1.17	0.51-2.65	0.71
**Atherosclerotic risk factors**						
Diabetes Mellitus	0.90	0.54-1.51	0.70	0.95	0.51-1.77	0.88
Dyslipidemia	1.61	0.89-2.87	0.11			
Hypertension	0.64	0.34-1.16	0.15	0.48	0.23-1.01	0.053
Smoking history	1.87	1.12-3.14	0.017	1.77	0.95-3.33	0.074
Chronic kidney disease	1.08	0.63-1.87	0.79	0.61	0.31-1.21	0.16
**Comorbidities**						
Old myocardial infarction	1.05	0.61-1.81	0.87			
Ischemic stroke	0.77	0.35-1.74	0.51			
Peripheral artery disease	2.93	0.96-12.8	0.06			
Multivessel disease	1.42	0.80-2.60	0.24			
Polyvascular disease	1.52	0.77-3.20	0.23			
**Laboratory data**						
eGFR (ml/min/1.73m^2^)	0.99	0.98-1.00	0.21			
HbA1c (%)	0.95	0.74-1.23	0.69			
HDL-C (mg/dl)	1.01	0.99-1.03	0.53			
LDL-C (mg/dl)	1.01	0.999-1.01	0.092	1.01	0.996-1.01	0.23
Triglyceride (mg/dl)	1.00	0.999-1.00	0.32			
hs-CRP (mg/dl)	1.51	0.94-2.97	0.094	1.67	0.99-3.42	0.050
Lp(a) > 30 mg/dL	3.19	1.67-6.56	<0.001	3.56	1.63-7.80	0.002
**Medications**						
Aspirin	0.69	0.41-1.16	0.16			
Statin	0.73	0.40-1.30	0.29			

CI, confidence interval; eGFR, estimated glomerular filtration rate; HbA1c, hemoglobin A1c; HDL-C, high-density lipoprotein cholesterol; hs-CRP, high-sensitivity C-reactive protein; LDL-C, low-density lipoprotein cholesterol; Lp(a), lipoprotein(a); OR, odds ratio. Multivariable analysis adjusted for age, sex, diabetes mellitus, hypertension, smoking history, chronic kidney disease, LDL-C, and hs-CRP.

### 3.5 Representative case

Supplementary Fig. 5 shows representative angioscopic images obtained from a patient with elevated Lp(a) levels. Multiple vulnerable plaque morphologies, including plaque rupture, ulceration with thrombi, fissure, and yellow plaque, were observed throughout the entire aorta, demonstrating diffuse aortic plaque vulnerability.

## 4 Discussion

In the present study, elevated serum Lp(a) levels were significantly associated with vulnerable aortic plaque characteristics assessed by NOGA. Although previous coronary imaging studies have consistently linked elevated Lp(a) levels with vulnerable plaque phenotypes, data regarding aortic plaque vulnerability remain limited. The present findings extend these observations to the aorta through direct angioscopic visualization. Notably, the prevalence of yellow plaque was comparable between groups, whereas plaque rupture and thrombus formation were markedly more frequent among patients with elevated Lp(a) levels. Furthermore, elevated Lp(a) levels remained independently associated with plaque rupture after adjustment for traditional cardiovascular risk factors. These findings suggest that elevated Lp(a) levels may be associated not only with atherosclerotic plaque burden, but more importantly with plaque destabilization and rupture in the aorta.

Elevated serum Lp(a) levels are well known to be associated with increased risk of ASCVD, including myocardial infarction, ischemic stroke, peripheral artery disease, and cardiovascular death [[5], [6], [7], [8]]. In the present study, patients with elevated Lp(a) levels more frequently had a history of myocardial infarction, peripheral artery disease, and polyvascular disease, supporting the association between elevated Lp(a) levels and systemic atherosclerotic disease burden. Lp(a) is considered to possess highly proatherogenic properties. Mendelian randomization analyses comparing the atherogenicity of LDL and Lp(a) particles have suggested that Lp(a) particles may have substantially greater atherogenic potential than LDL particles [18]. Previous studies evaluating the relationship between Lp(a) and aortic atherosclerosis have primarily focused on plaque burden, aortic atheroma severity, or vascular calcification. In contrast, the association between Lp(a) and vulnerable aortic plaque characteristics remains insufficiently investigated [[19], [20], [21]]. The present findings extend these observations by demonstrating the association between elevated Lp(a) levels and vulnerable aortic plaque characteristics assessed by direct angioscopic visualization. The consistency of these findings using both the conventional 30 mg/dL cutoff and the internationally recognized 50 mg/dL cutoff further supports the robustness of the observed association.

Lp(a) may also be associated with plaque characteristics and plaque destabilization. Previous coronary imaging studies using multiple imaging modalities have consistently demonstrated associations between elevated Lp(a) levels and vulnerable plaque phenotypes. An IVUS study using radiofrequency analysis demonstrated that elevated Lp(a) levels were associated with a larger necrotic core component [9]. NIRS-IVUS studies have shown an association between Lp(a) levels and lipid-rich plaque burden [22]. OCT studies have demonstrated associations between elevated Lp(a) levels and thin-cap fibroatheroma [23]. CCTA studies have similarly demonstrated associations with high-risk plaque features, including low attenuation and positive remodeling [24]. Importantly, a substudy of the PROSPECT II trial demonstrated that whereas elevated LDL-C levels were associated with diffuse plaque burden and lipid deposition throughout the coronary artery tree, elevated Lp(a) levels were more closely associated with focal high-risk vulnerable plaques responsible for future acute coronary syndromes [25]. The present findings extend these observations to the aorta through direct angioscopic visualization. In contrast to the similar prevalence of yellow plaque between groups, elevated Lp(a) levels were strongly associated with plaque rupture and thrombus formation, suggesting a closer relationship with plaque destabilization rather than simple plaque burden. The lack of association between Lp(a) levels and yellow plaque prevalence, despite strong associations with plaque rupture and thrombus formation, further suggests that Lp(a) may be more closely linked to plaque destabilization than to plaque burden alone. The association remained significant when Lp(a) was analyzed as a continuous variable, supporting a graded association between increasing Lp(a) levels and plaque rupture. Consistent findings were also observed using the internationally accepted Lp(a) cutoff of >50 mg/dL, and the prevalence of plaque rupture increased progressively across Lp(a) categories, further supporting a dose-dependent relationship between Lp(a) levels and aortic plaque vulnerability. Importantly, direct angioscopic visualization enables assessment of plaque rupture and thrombus formation, findings that cannot be readily evaluated using conventional imaging modalities.

The relationship between Lp(a) and cardiovascular risk appears to be largely independent of LDL-C levels. A participant-level meta-analysis demonstrated that elevated Lp(a) levels remained associated with ASCVD events across achieved LDL-C levels, including among patients with low LDL-C levels following statin therapy [26]. Furthermore, Lp(a) has been associated with the severity of thoracic aortic atherosclerosis independently of LDL-C and other conventional risk factors [20]. In the present study, elevated Lp(a) levels remained associated with plaque rupture even among patients who achieved LDL-C levels <70 mg/dL. Importantly, this association was also preserved among patients achieving the more stringent LDL-C target of <55 mg/dL, suggesting that Lp(a)-related plaque vulnerability persists despite intensive LDL-C lowering. These findings suggest that Lp(a)-related residual risk may be mediated, at least in part, through plaque destabilization and rupture, rather than through LDL-C–related plaque burden alone.

Lp(a) is a representative genetically determined ASCVD risk factor, and approximately one in five individuals worldwide is reported to have elevated Lp(a) levels [2,27]. Ethnic differences in Lp(a) distribution have also been reported, and approximately one in ten of the general Asian population may have Lp(a) levels >50 mg/dL [28]. This prevalence is substantially higher than the estimated prevalence of familial hypercholesterolemia, which is approximately 1 in 300 individuals [29]. In the present study, markedly elevated Lp(a) levels >50 mg/dL were observed in approximately 15% of patients with coronary artery disease, and these patients showed a particularly high prevalence of plaque rupture. Together with the observed graded association between Lp(a) levels and plaque rupture, these findings suggest that elevated Lp(a) may identify patients with increased aortic plaque vulnerability. The primary analyses were performed using a cutoff value of 30 mg/dL, which has been adopted in Japanese clinical practice and previous epidemiological studies [30,31]. Importantly, consistent findings were also obtained using the internationally accepted cutoff value of 50 mg/dL [32], further supporting the robustness and clinical relevance of the present findings. At present, cardiovascular outcome benefits of selective Lp(a)-lowering therapy have not yet been established. Several Lp(a)-lowering therapies are currently under development, and ongoing phase 3 cardiovascular outcome trials will determine whether pharmacological Lp(a) reduction translates into improved cardiovascular outcomes [33,34]. Nevertheless, intensive management of modifiable cardiovascular risk factors is recommended to reduce overall ASCVD risk, even in individuals with elevated Lp(a) levels [32,35,36]. In this context, the European Atherosclerosis Society consensus statement recommends intensified management of cardiovascular risk factors according to Lp(a) levels and overall cardiovascular risk. Furthermore, approaches have been proposed to estimate the additional LDL-C reduction required to mitigate the excess cardiovascular risk associated with elevated Lp(a) levels, further emphasizing the clinical importance of identifying elevated Lp(a) [35].

The present findings suggest that elevated Lp(a) levels may be associated with plaque destabilization and rupture even in patients who achieved LDL-C levels <70 mg/dL, including those achieving the current guideline-recommended LDL-C target of <55 mg/dL, highlighting the potential importance of comprehensive residual risk management in patients with elevated Lp(a) levels.

This study has several limitations. First, this was a single-center study with a relatively limited sample size. Second, all study participants had established coronary artery disease and underwent clinically indicated catheterization and NOGA, which may have introduced selection bias. Therefore, the present findings may not be generalizable to broader populations. Third, this study was cross-sectional in nature, and clinical outcomes were not evaluated. Accordingly, the relationship between elevated Lp(a) levels, plaque rupture, and future cardiovascular events remains uncertain. Although multivariable adjustment was performed, patients with elevated Lp(a) levels had a higher burden of systemic atherosclerotic disease; therefore, residual confounding related to overall disease severity cannot be excluded. Finally, NOGA is currently available only in specialized centers and is not routinely used in contemporary clinical practice.

## 5 Conclusion

The present findings suggest that elevated Lp(a) levels may be associated with plaque destabilization and rupture, even among patients achieving contemporary LDL-C targets, highlighting the potential importance of comprehensive residual risk management. Further prospective studies are warranted to confirm these findings.

## Ethics declaration

Written informed consent to take part in the study and to publish the article has been obtained from all participants or their legal representatives. The privacy rights of participants have been observed.

This study was performed in compliance with relevant laws, regulatory frameworks and guidelines where the research took place.This study was approved by the Nihon University Itabashi Hospital.(Approval No. RK-210713-7)

## Author contributions

K.K. contributed to the study conception and design, data acquisition, data analysis, interpretation of data, and drafting of the manuscript. Y.T., K.F., Y.E., T.N., N.K., S.T., Y.H., T.N., R.S., S.M., S.M., M.M., K.H., Y.K., R.A., S.M., M.S., and D.F. contributed to data acquisition and interpretation of data. Y.O. supervised the study and critically revised the manuscript for important intellectual content. All authors reviewed and approved the final manuscript.

## Funding

This work was supported by 10.13039/501100001691JSPS KAKENHI Grant Number 21K16041.

## Declaration of competing interest

The authors declare the following financial interests/personal relationships which may be considered as potential competing interests:K.K. has received lecture fees from Amgen K.K. and Novartis Pharma K.K. and research funding from Nipro Corporation. Y.O. reports honoraria, research funding, lecture fees, and/or endowed chair support from pharmaceutical and medical device companies, including AstraZeneca K.K., Johnson & Johnson/Biosense Webster, Medtronic Japan Co., Ltd., Bayer Yakuhin, Ltd., Abbott Medical Japan LLC, Boston Scientific Japan K.K., Daiichi Sankyo Co., Ltd., and Pfizer Japan Inc. The remaining authors declare no conflicts of interest.
