# Supplementary material for: Association between serum lipoprotein(a) levels and aortic vulnerable plaques assessed by non-obstructive general angioscopy
Source: Atheroscler Plus. 2026 Sep 12;66:100603. doi: 10.1016/j.athplu.2026.100603 (PMC13594957; doi:10.1016/j.athplu.2026.100603)
Supplement: Supplementary file 1 [file mmc1.docx]

**Supplementary Figure 1. Study flow chart**

Lp(a), lipoprotein (a).

**Supplementary Figure 2. Distribution of serum Lp(a) levels**

Twenty-seven percent of patients had serum Lp(a) levels >30 mg/dL, and 14% had markedly elevated Lp(a) levels >50 mg/dL.

Lp(a), lipoprotein(a)

**Supplementary Figure 3. Association between plaque characteristics and serum Lp(a) levels among patients with LDL-C <70 mg/dL.**

Elevated serum Lp(a) levels (>30 mg/dL) remain associated with a higher prevalence of plaque rupture and thrombus among patients with LDL-C <70 mg/dL.

LDL-C, low-density lipoprotein cholesterol; Lp(a), lipoprotein(a)

**Supplementary Figure 4. Association between plaque characteristics and serum Lp(a) levels among patients with LDL-C <55 mg/dL.**

Elevated serum Lp(a) levels (>30 mg/dL) remain associated with a higher prevalence of plaque rupture among patients with LDL-C <55 mg/dL.

LDL-C, low-density lipoprotein cholesterol; Lp(a), lipoprotein(a)

**Supplementary Figure 5. Representative case**

Representative angioscopic findings in a 79-year-old man with elevated serum Lp(a) levels (50 mg/dL). Despite lipid-lowering therapy with atorvastatin 20 mg and ezetimibe 10 mg, and controlled LDL-C levels (68 mg/dL), multiple vulnerable plaque morphologies are observed throughout the entire aorta, including plaque rupture (A, C, and E), thrombus (B and E), and yellow plaque (D).

LDL-C, low-density lipoprotein cholesterol; Lp(a), lipoprotein(a)

**Supplementary Table 1**

LDL-C, low-density lipoprotein cholesterol; Lp(a), lipoprotein(a)

**Supplementary Table 2**

CI, confidence interval; eGFR, estimated glomerular filtration rate; HbA1c, hemoglobin A1c; HDL-C, high-density lipoprotein cholesterol; hs-CRP, high-sensitivity C-reactive protein; LDL-C, low-density lipoprotein cholesterol; Lp(a), lipoprotein(a); OR, odds ratio. Multivariable analysis adjusted for age, sex, diabetes mellitus, hypertension, smoking history, chronic kidney disease, LDL-C, and hs-CRP.

**Supplementary Table 3**

CI, confidence interval; eGFR, estimated glomerular filtration rate; HbA1c, hemoglobin A1c; HDL-C, high-density lipoprotein cholesterol; hs-CRP, high-sensitivity C-reactive protein; LDL-C, low-density lipoprotein cholesterol; Lp(a), lipoprotein(a); OR, odds ratio. The same multivariable model as Table 2 was used, except that Lp(a) was entered as a continuous variable.

**Supplementary Table 4**

CI, confidence interval; eGFR, estimated glomerular filtration rate; HbA1c, hemoglobin A1c; HDL-C, high-density lipoprotein cholesterol; hs-CRP, high-sensitivity C-reactive protein; LDL-C, low-density lipoprotein cholesterol; Lp(a), lipoprotein(a); OR, odds ratio. The analysis was restricted to male patients (n=224). The multivariable model included age, diabetes mellitus, hypertension, smoking history, chronic kidney disease, LDL-C, hs-CRP, and elevated Lp(a) (>30 mg/dL).
